# Harnessing innate immunity against glioblastoma microenvironment

**DOI:** 10.3389/fimmu.2025.1648601

**Published:** 2025-07-25

**Authors:** Wenbo Zhang, Wanhong Zhang, Henghao Wu, Xinsheng Han

**Affiliations:** ^1^ Department of Neurology, Kaifeng Central Hospital, Xinxiang Medical University, Kaifeng, China; ^2^ Department of Neurosurgery, Kaifeng Central Hospital, Kaifeng, China; ^3^ Department of Neurology, Kaifeng Central Hospital, Kaifeng, China; ^4^ Henan Key Laboratory of Neuromuscular Pathology, Kaifeng Central Hospital, Kaifeng, China

**Keywords:** glioblastoma, innate immunity, tumor-associated macrophages, NK cell, microglia, myeloidderived suppressor cells, tumor microenvironment, immunotherapy

## Abstract

Glioblastoma (GBM) possesses a profoundly immunosuppressive tumor microenvironment (TME) dominated by innate immune mechanisms. Tumor-associated macrophages (TAMs), microglia, and myeloid-derived suppressor cells (MDSCs) constitute the major immunosuppressive axis, promoting tumor progression through cytokine secretion (IL-10, TGF-β), metabolic reprogramming, and inhibition of cytotoxic immunity. These innate immune cells not only facilitate immune evasion but also impair adaptive T-cell responses, limiting the efficacy of current immunotherapies. Emerging evidence highlights the therapeutic potential of targeting innate immunity via TAM repolarization, MDSC depletion, and NK cell activation to reshape the immunosuppressive TME. This review summarizes the pivotal role of innate immunity in GBM pathogenesis and explores novel combinatorial strategies that integrate innate immune modulation with checkpoint blockade, oncolytic virotherapy, and metabolic interventions to overcome therapeutic resistance in this lethal malignancy.

## 1 Introduction

Glioblastoma, the most common primary central nervous system (CNS) malignancy, accounts for 80% of primary malignant brain tumors (1). Although molecular advances have enhanced our understanding of GBM pathogenesis and led to improved treatment strategies, substantial gaps remain in translating these discoveries into effective clinical interventions (2–4). Despite advancements in surgical resection, radiation therapy optimization, and molecularly targeted treatments, clinical outcomes remain suboptimal. Consequently, there is a pressing demand for innovative therapeutic modalities and novel pharmacological interventions.

Cancer immunotherapy, particularly immune checkpoint inhibition, has revolutionized oncology by facilitating durable antitumor immune responses (5). Clinically, inhibitors targeting PD-1/PD-L1 and CTLA-4 have demonstrated efficacy in malignancies such as melanoma, non-small cell lung carcinoma, and renal cell cancer. However, their effectiveness in GBM remains constrained due to the unique immunosuppressive tumor microenvironment and sophisticated immune escape mechanisms (6–8). These findings indicate that monotherapy failure results from multifactorial mechanisms, including the accumulation of immunosuppressive cell subsets, dysregulated cytokine signaling, and impaired antigen presentation within the GBM microenvironment (9–11). Moreover, GBM cells utilize multifaceted immune evasion strategies, such as overexpressing immunosuppressive ligands and recruiting Tregs and MDSCs, which collectively undermine therapeutic efficacy (12). Consequently, comprehensive characterization of the glioma immune landscape and intricate cellular crosstalk within the tumor microenvironment is essential for developing optimized, patient-specific immunotherapeutic strategies.

## 2 Glioblastoma microenvironment

### 2.1 Innate immunity

#### 2.1.1 Tumor-associated macrophages in glioma

Within the glioma immune landscape, TAMs represent the predominant immune population, consisting of both bone marrow-derived macrophages (BMDMs) and resident microglial cells (13, 14). Microglia arise from primitive yolk sac precursors and maintain their population through CSF1R-dependent self-renewal, whereas BMDMs are recruited from peripheral circulation primarily via the CCL2/CCR2 chemotactic axis (15). Historically, these subsets were distinguished by surface markers—CD11b^+^CD45^high for macrophages versus CD11b^+^CD45^low for microglia (16)—though recent single-cell transcriptomic studies have identified more refined signatures, including CCR2, CD45RA, and CD209 for macrophages, and CX3CR1, P2RY12, and TMEM119 for microglia (17). Morphologically, microglia display an extended, ramified structure, while macrophages appear more compact and highly motile (18). Although microglia serve as the primary immune sentinels in the CNS, BMDMs constitute the majority of TAMs in GBM, with microglia predominating in primary tumors and macrophages increasing in recurrent disease (16, 19). Elevated TAM infiltration is strongly associated with worse clinical outcomes. The functional plasticity of TAMs is a key focus in neuroimmunology (20).

Conventionally, these cells are categorized into M1 and M2 phenotypes. M1 macrophages are pro-inflammatory, induced by interferon-gamma and tumor necrosis factor-alpha, characterized by the expression of CD80 and CD86, and secrete reactive oxygen species, interleukin-1β, and interleukin-12. In contrast, M2 macrophages exhibit immunosuppressive properties, are polarized by interleukin-4 and interleukin-13, express CD206 and arginase-1, and produce interleukin-10, transforming growth factor-beta, and CCL17 (21, 22). However, high-resolution single-cell analyses have revealed considerable transcriptional overlap, which challenges the traditional binary classification and instead supporting a continuum of activation states (23). TAMs facilitate immune escape through multiple mechanisms, including MET–STAT4–PD-L1 signaling (24) and TLX-induced PD-L1 upregulation, which suppresses tumor-infiltrating lymphocytes (TILs) (25). Additionally, glioma-derived exosomes promote lipid accumulation in macrophages via TMEM198B, reinforcing M2-like polarization (26). Given the critical role of TAM heterogeneity in disease progression, personalized immunotherapeutic approaches targeting immunosuppressive TAM subsets may enhance treatment responses in glioma patients.

#### 2.1.2 Neutrophils and NK cells in the glioblastoma microenvironment

As critical effectors of innate immunity, neutrophils represent nearly 70% of peripheral leukocytes and play context-dependent roles in glioblastoma pathogenesis (27). During initial tumor development, these cells exert antitumor effects by secreting antimicrobial peptides and pro-inflammatory cytokines, thereby enhancing immune-mediated tumor suppression. However, in advanced disease stages, the TME reprograms neutrophil activity, shifting their function toward promoting tumor expansion and metastatic dissemination (28). Within the TME, neutrophils engage in extensive crosstalk with other immune populations, secreting a variety of soluble factors that facilitate neoplastic growth, vascular remodeling, and the establishment of an immunosuppressive niche (29). NK cells, another vital component of innate immunity, are widely present in the GBM microenvironment, though their tumoricidal capacity is often severely attenuated (30). Malignant cells employ numerous immune escape mechanisms, including reduced expression of activating ligands and increased presentation of inhibitory signals, which collectively impair NK cell recognition and cytotoxic function (31). Additionally, immunosuppressive cytokines such as TGF-β and IL-10 within the TME further inhibit NK cell activity, reinforcing immune evasion. Nevertheless, NK cells retain significant therapeutic potential, as they can selectively target and eliminate treatment-resistant glioma stem cells while also enhancing antitumor immunity through mechanisms like antibody-dependent cellular cytotoxicity (ADCC) (32–34).

### 2.2 Myeloid-derived suppressor cells and dendritic cells

MDSCs constitute a diverse group of immature myeloid lineage cells exhibiting strong immunosuppressive properties. Increased MDSC populations have been observed in the circulation and tumor microenvironments of individuals with glioblastoma (35). These cells promote oncogenesis through multiple mechanisms, including the release of immunomodulatory cytokines and growth factors, inhibition of antitumor immune effector functions, and stimulation of vascular proliferation. Notably, IL-6 production by MDSCs is mediated through STAT3 pathway activation, which supports neoplastic cell growth and metastatic behavior (36). Moreover, MDSCs enhance PD-L1 expression, resulting in diminished natural killer cell and T lymphocyte function while reinforcing the immunosuppressive characteristics of the tumor niche (37). As the most efficient professional antigen-presenting cells, DCs play a pivotal role in priming naïve T cells and orchestrating adaptive immunity (38). Within GBM, overexpression of macrophage migration inhibitory factor (MIF) facilitates disease progression by triggering autophagic processes and impairing DC-mediated immune recognition (39). Furthermore, the oncogenic protein annexin A1, which is highly expressed in glioblastoma, disrupts DC differentiation via NF-κB-dependent mechanisms. This leads to elevated IL-8 secretion and p65 phosphorylation, collectively promoting tumor immune evasion (40). Additionally, tumor-derived PGE2 contributes to immune suppression by inhibiting dendritic cell maturation through EP2 and EP4 receptor signaling. Activation of these G protein–coupled receptors elevates intracellular cAMP levels and downstream PKA activity, thereby reducing MHC class II expression and costimulatory molecules such as CD80 and CD86 (41). This impaired maturation limits the antigen-presenting capacity of DCs and blunts the priming of cytotoxic T lymphocytes, further facilitating immune evasion in the GBM microenvironment.

### 2.3 Microglia

Microglia serve as the principal immunocompetent cells within the central nervous system, maintaining a quiescent surveillance state characterized by branched processes during homeostasis, yet rapidly transitioning to an activated phenotype upon encountering pathological triggers to execute immunoprotective functions (42). Within glioma ecosystems, these neural immune effectors migrate toward tumor masses guided by chemoattractant molecules including CCL2, subsequently secreting an array of soluble mediators such as interleukin 6, transforming growth factor beta, and vascular endothelial growth factor that collectively promote neoplastic invasion and expansion (43). The immune architecture of gliomas emerges from dynamic intercellular communication among diverse leukocyte populations. Of particular significance, tumor infiltrating macrophages generate immunosuppressive factors including transforming growth factor beta and interleukin 10 that simultaneously dampen CD8^+^ T cell effector mechanisms while stimulating regulatory T cell clonal expansion, thereby cultivating an immunotolerant microenvironment (12). Additionally, these macrophage populations overexpress coinhibitory molecules including programmed death ligand 1 that directly compromise T cell antitumor capacity. The synergistic action of immunosuppressive mediators originating from both neoplastic cells and tumor associated macrophages promotes T cell hyporesponsiveness, ultimately subverting antitumor immunity during glioma pathogenesis (44). The programmed death 1 programmed death ligand 1 axis constitutes a fundamental pathway facilitating glioma immune evasion. Neoplastic cells increase programmed death ligand 1 surface expression to engage programmed death 1 receptors on T lymphocytes, resulting in functional inhibition and progressive exhaustion that enables tumor immune escape. This immunosuppressive cascade becomes further intensified through the combined action of inhibitory cytokines including transforming growth factor beta and interleukin 10 together with regulatory immune cell populations such as tumor associated macrophages and regulatory T cells, ultimately generating a sophisticated and multilayered immune suppression network (45).

### 2.4 Adaptive immunity

Within the tumor microenvironment of GBM, CD4^+^ and CD8^+^ T lymphocytes constitute the dominant adaptive immune cell subsets, comprising roughly 5% of all CD45^+^ leukocytic infiltrates (46). Notably, GBM with wild-type isocitrate dehydrogenase (IDH) status demonstrate greater T cell infiltration compared to their IDH-mutant counterparts (47). Despite their presence, these T cells often undergo functional exhaustion, primarily driven by chronic exposure to tumor-associated antigens. This exhausted phenotype is characterized by reduced proliferation, compromised cytotoxic activity, and elevated expression of immune-inhibitory molecules, including PD-1 and CTLA-4 (48). Furthermore, immunosuppression within GBM is amplified by CD4^+^CD25^+^FoxP3^+^ regulatory T cells (Tregs), which actively suppress antitumor immunity (49). While cytotoxic T cells retain intrinsic tumoricidal capacity, their efficacy is substantially hindered in the GBM TME due to heightened immune checkpoint signaling and the prevalence of immunosuppressive cellular populations (50). Despite constituting a relatively small fraction of immune infiltrates in GBM, B lymphocytes play a pivotal role in modulating tumor biology and treatment outcomes (51). The B cell compartment within GBM includes both immunoregulatory B cells (Bregs) that suppress immune responses and conventional B lymphocytes capable of antigen presentation, which can amplify T cell activation (52, 53). These cells mediate immunosuppressive effects primarily through the production of IL-10 and TGF-β, cytokines that impair the cytotoxic function of T cells and NK cells while simultaneously promoting processes associated with neural development and tumor infiltration (54). Additionally, B cells contribute to tumor vascularization by secreting angiogenic factors, including VEGF, CXCL12, and CXCL13. These molecules foster the formation of new blood vessels, thereby enhancing oxygen and nutrient delivery to support tumor growth (**Table 1**) (54).

**Table 1:** Immune cells in the GBM microenvironment.

Cell type	Markers	Key function	Immunosuppressive mechanisms	Treatment
TAMs	Microglia (TMEM119^+^, P2RY12^+^); BMDMs (CCR2^+^, CD45^+^high)	ECM remodeling, angiogenesis, PD-L1 induction, immunosuppression	IL-10, TGF-β, PD-L1, M2 polarization, STAT3 activation	CSF1R inhibition, PD-L1 blockade, TAM reprogramming
T Lymphocytes (CD4^+^, CD8^+^, Tregs)	CD3^+^, CD4^+^/CD8^+^; Tregs: CD25^+^FoxP3^+^	Cytotoxicity, immunoregulation, tolerance induction	PD-1, CTLA-4 upregulation, Treg suppression	Checkpoint inhibitors, Treg depletion
Neutrophils	CD11b^+^Ly6G^+^	Early anti-tumor effects; later promote angiogenesis, invasion	Reprogramming by TME signals; cytokine release	CXCR2 blockade, inhibit late-phase polarization
B Cells	CD19^+^, CD20^+^; Bregs	IL-10 and VEGF secretion, antigen presentation	TGF-β and IL-10 suppress effector immunity; promote angiogenesis	Anti-VEGF/IL-10 therapy, B cell modulation
NK Cells	CD56^+^CD3^-^; NKp46^+^, NKG2D^+^, CD16^+^	Kill tumor & GSCs, mediate ADCC	Downregulation of activating ligands; suppression by TGF-β, IL-10	TGF-β blockade, ligand expression restoration
MDSCs	CD11b^+^, Gr1^+^, HLA-DR^-^	Suppress T/NK cells, secrete IL-6, promote angiogenesis	STAT3 activation, PD-L1/IL-6 production	STAT3/IL-6 inhibition, anti-MDSC therapies
Dendritic Cells	CD11c^+^MHC-II^+^, CD80^+^, CD86^+^	Antigen presentation, T cell priming	MIF, Annexin A1 impair maturation, induce IL-8 and p65 activation	DC vaccines, maturation stimulants
Microglia	TMEM119^+^, Iba1^+^, CX3CR1^+^	Secrete IL-6, VEGF, TGF-β; support invasion, regulate local immunity	Promote Treg expansion, PD-L1 expression, release immunosuppressive cytokines	Reprogramming, M1 activation, checkpoint inhibition

## 3 Biological mechanisms underlying the GBM microenvironment

Glioblastoma represents a complex biological system where diverse cell types and molecular mediators interact through intricate signaling networks, driving malignancy and therapeutic resistance. A hallmark of GBM is aberrant angiogenesis, primarily driven by elevated expression of angiogenic mediators such as VEGF, bFGF, HGF, PDGF, TGF-β, MMPs, and angiopoietins. These factors are upregulated through oncogene activation, tumor suppressor loss, and hypoxia-induced stress responses (55, 56). FGFRs promote neovascularization via activation of PI3K/AKT/mTOR and c-JUN/p38-MAPK/STAT3/NF-κB pathways, facilitating tumor growth and vascular development (57, 58). The extracellular matrix (ECM) supports vascular expansion, tumor infiltration, and resistance to therapy (59). Key ECM components include fibronectin-C, which enhances cellular invasiveness (60), fibronectin, which contributes to chemoresistance (61), Fibulin-3, which activates Notch/NF-κB and induces IL-6 secretion from TAMs through integrin αvβ3/FAK signaling (62), and hyaluronic acid, which accelerates cell migration (61). Metabolic reprogramming and immune regulation are tightly linked in GBM. Lactate accumulation acidifies the microenvironment, impairing cytotoxic T and NK cell function while expanding Tregs and MDSCs (63). Beyond acidification, lactate activates GPR81 on TAMs, suppressing NF-κB signaling and promoting an immunosuppressive TAM phenotype. TAM-derived IL-10 and TGF-β drive metabolic shifts toward glycolysis and lipid synthesis, enhancing tumor cell survival (64). Targeting lactate reduces immune evasion and genomic repair via XRCC1 lactylation blockade, improving treatment efficacy (65). Moreover, enzymes such as IDO co-express with immune checkpoints like PD-L1, reinforcing immune tolerance (66). Cytokines and chemokines mediate bidirectional communication between GBM cells and immune elements. IL-6 promotes tumor proliferation, while TGF-β and IL-10 enhance Treg-mediated immunosuppression (67). Tumor-derived exosomes carrying PD-L1 and proteases reshape the microenvironment and facilitate metastasis (68). Hypoxia activates HIFs, promoting cell proliferation, angiogenesis, and metabolic adaptation (69), while also impairing NK and effector T cell function and enriching immunosuppressive cells such as Tregs and MDSCs (70, 71). The convergence of vascular remodeling, ECM dynamics, metabolic adaptation, and immune evasion underscores the complexity of the GBM niche and its role in therapy resistance.

## 4 Tumor microenvironment-targeted therapies in GBM

### 4.1 Immune checkpoint inhibitors

**Figure 1:**
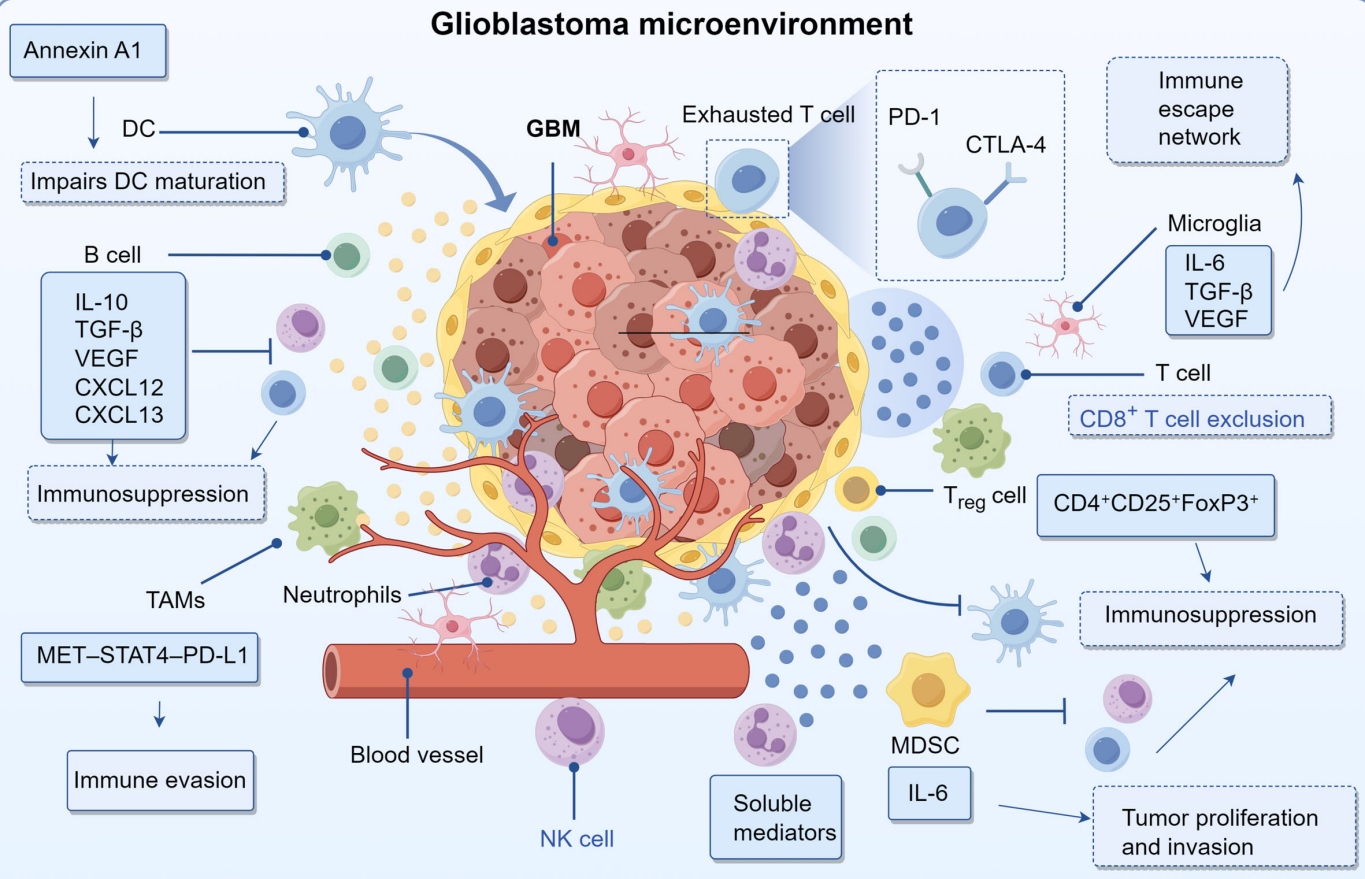
Mechanism of immune cells in GBM progression.

Immunological checkpoint molecules, primarily expressed on immune effector cells—especially T lymphocytes—are essential for maintaining self-tolerance and preventing autoimmune responses. However, malignant cells frequently hijack these regulatory pathways to evade immune-mediated elimination (72). Checkpoint blockade therapeutics reinvigorate the cytotoxic potential of T cells. Cytotoxic T-lymphocyte-associated protein 4 (CTLA-4), the initial clinically validated immune checkpoint, attenuates T cell stimulation by outcompeting CD28 for binding to CD80/CD86 ligands on antigen-presenting cells. Clinical evaluation of ipilimumab, an antagonist of CTLA-4, in patients with glioblastoma revealed no improvement in outcomes compared to temozolomide in Phase II clinical trials (73). The PD-1/PD-L1 signaling axis mediates T cell suppression, with elevated PD-L1 expression serving as a negative prognostic indicator (74–76). CD47 interaction with SIRPα on phagocytic cells prevents tumor cell engulfment, thereby driving disease recurrence; disruption of this molecular interaction could potentiate checkpoint immunotherapy (77, 78). Elevated TIM-3 levels in GBM correlate with enhanced tumor aggressiveness (79, 80), whereas indoleamine 2,3-dioxygenase (IDO) suppresses cytotoxic lymphocyte function and shows increased activity in GBM specimens (81). Pharmacological inhibition of IDO, such as through the use of epacadostat, has shown promising results in preclinical studies, particularly when combined with radiotherapy or anti-PD-1 immunotherapy (82). The limited clinical efficacy of immune checkpoint inhibitors in glioblastoma is multifactorial. One contributing mechanism is the immunosuppressive tumor microenvironment, characterized by the secretion of interleukin-10 and transforming growth factor-beta by M2-polarized tumor-associated macrophages and myeloid-derived suppressor cells, which collectively suppress cytotoxic immune responses (83). Additionally, the inherently low tumor mutational burden in glioblastoma results in a scarcity of immunogenic neoantigens, thereby reducing the likelihood of effective immune recognition (84). Furthermore, the structural and functional integrity of the blood-brain barrier restricts the intratumoral delivery of immunotherapeutic agents, further limiting clinical benefit. Recent studies have demonstrated that galectin-9, secreted by glioblastoma stem-like cells, engages the TIM-3 receptor on Th1 lymphocytes, contributing to their functional exhaustion and promoting immune evasion (85). This interaction activates intracellular apoptotic signaling cascades, leading to Th1 cell apoptosis, while concurrently suppressing the production of key cytokines such as interleukin-2 and interferon-gamma. The resulting impairment of Th1-mediated immune responses facilitates the establishment of an immunosuppressive tumor microenvironment conducive to glioblastoma progression (**Figure 1**) (86).

### 4.2 Chimeric antigen receptor T cell therapy

As a groundbreaking form of immunotherapy, CAR-T cell therapy involves genetically engineering autologous T lymphocytes to express synthetic receptors that recognize tumor-associated antigens independently of MHC restriction. Despite this innovation, its efficacy against GBM remains limited, with median overall survival (mOS) reaching only 8 months for EGFRvIII-targeted therapy and 11.1 months for HER2-directed approaches, underscoring challenges posed by tumor invasiveness and immune evasion (87). Key challenges include: (1) antigenic heterogeneity enabling immune escape; (2) an immunosuppressive microenvironment enriched with MDSCs and Tregs; (3) poor CAR-T cell trafficking across the BBB; and (4) antigen loss through clonal selection. Recent studies demonstrate that trogocytosis mediates CAR molecule transfer to tumor cells, a resistance mechanism modulated by antigen density, receptor affinity, and lipid metabolism (87). Current research focuses on optimizing CAR activation thresholds to adapt to heterogeneous antigen expression.

### 4.3 Oncolytic virotherapy

A novel immunotherapeutic approach for GBM involves the use of oncolytic viruses, which are genetically modified to preferentially infect and propagate within malignant cells, leading to their destruction (88). This process triggers the release of danger-associated molecular patterns (DAMPs) along with proinflammatory cytokines, transforming immunologically inert (“cold”) tumors into immunologically active (“hot”) lesions (89). Among the earliest viruses adapted for GBM therapy is herpes simplex virus (HSV), with modified variants including HSV-1716, G207, and G47Δ demonstrating favorable safety profiles and selective tumor tropism in preclinical and early clinical studies (90, 91). Additionally, PVSRIPO—a recombinant poliovirus-rhinovirus chimera—has been developed to target CD155, a receptor frequently upregulated in GBM (92). In a Phase I clinical trial (NCT01491893), intratumoral administration of PVSRIPO in recurrent GBM patients was well tolerated, yielding a median overall survival (mOS) of 12.5 months—exceeding the historical benchmark of 11.3 months. Furthermore, survival rates at 24 and 36 months reached 21%, significantly higher than those observed in control groups (14% and 4%, respectively) (93). Ongoing investigations include a Phase II monotherapy trial (NCT02986178) as well as combination studies incorporating immune checkpoint inhibitors such as atezolizumab (anti–PD-L1) or pembrolizumab (anti–PD-1) (NCT03973879, NCT04479241), aiming to evaluate potential synergistic effects.

### 4.4 Cancer vaccines

Designed to elicit adaptive immunity targeting tumor-associated or tumor-specific antigens, cancer vaccines represent a promising strategy for GBM treatment (94). These immunogens can originate from endogenous tumor-derived proteins or exogenous pathogens such as cytomegalovirus (CMV) (95, 96). Some formulations incorporate predefined antigens, whereas others rely on antigen-presenting cell (APC) activation to process unidentified tumor epitopes (97). Upon delivery, APCs prime T cells, which subsequently infiltrate malignant tissue, triggering cytotoxic activity and potentially generating durable immunological memory. Current vaccine modalities explored for GBM comprise peptide-based formulations, dendritic cell (DC)-based vaccines, nucleic acid (DNA/RNA) vaccines, and viral vector-delivered immunogens. As specialized APCs, DCs internalize tumor antigens and traffic to lymphoid organs to stimulate T cell activation (98, 99). DC vaccination entails isolating patient-derived DCs, pulsing them with tumor antigens, and reinfusing them to induce tumor-specific T cell responses (100). Preclinical studies demonstrated that DC vaccines incorporating EGFRvIII-transfected glioma cells elicited potent antitumor immunity and increased survival. A Phase II clinical trial assessing DC vaccination in newly diagnosed GBM patients reported enhanced modest overall survival benefits (101). Pooled analyses indicate that DC-based immunization substantially improves 1- and 2-year OS rates in treatment-naïve GBM cases (102). Furthermore, autologous tumor lysate-loaded DC vaccines conferred survival advantages in both newly diagnosed and recurrent GBM relative to conventional therapy, particularly benefiting MGMT-methylated subgroups (103).

## 5 Conclusion

Glioblastoma (GBM) presents a formidable therapeutic challenge due to its profoundly immunosuppressive TME, which is predominantly shaped by innate immune mechanisms. TAMs, MDSCs, and microglia establish an immunosuppressive niche through multiple mechanisms, including cytokine secretion (IL-10, TGF-β), metabolic reprogramming, and immune checkpoint upregulation. These cells not only facilitate tumor progression but also actively suppress adaptive immune responses, rendering conventional immunotherapies largely ineffective. The complexity of these interactions underscores the need for novel strategies that specifically target innate immune pathways to disrupt the immunosuppressive network and restore antitumor immunity.

Emerging therapeutic approaches, such as TAM repolarization, MDSC depletion, and NK cell activation, show promise in reshaping the GBM microenvironment. However, their full potential will likely be realized only when combined with other modalities, including immune checkpoint blockade, metabolic interventions, and precision targeting. Future research should focus on elucidating the intricate crosstalk between innate and adaptive immunity while developing integrated treatment strategies that simultaneously overcome immunosuppression and enhance tumor-specific immune responses. A paradigm shift toward innate immune-focused therapies, within a comprehensive multimodal framework, may finally break the therapeutic impasse in this.
